# Actuators with Two Double Gimbal Magnetically Suspended Control Moment Gyros for the Attitude Control of the Satellites

**DOI:** 10.3390/mi15091159

**Published:** 2024-09-16

**Authors:** Romulus Lungu, Alexandru-Nicolae Tudosie, Mihai-Aureliu Lungu, Nicoleta-Claudia Crăciunoiu

**Affiliations:** 1 Faculty of Electrical Engineering, University of Craiova, 200585 Craiova, Romania; romulus_lungu@yahoo.com (R.L.); lma1312@yahoo.com (M.-A.L.); efrimclaudia@gmail.com (N.-C.C.); 2 International Academy of Astronautics (IAA), 75016 Paris, France

**Keywords:** DGMSCMG, satellite, actuator, sensor, attitude, control

## Abstract

The paper proposes a novel automatic control system for the attitude of the mini-satellites equipped with an actuator having *N* = 2 DGMSCMGs (Double Gimbal Magnetically Suspended Control Moment Gyros) in parallel and orthogonal configuration, as well as a DGMSCMG-type sensor for the measurement of the satellite absolute angular rate. The proportional-derivative controller, designed based on the Lyapunov-functions theory, elaborates the control law according to which the angular rates applied to the servo systems for the actuation of the DGMSCMGs gyroscopic gimbals are computed. The gimbal’s angular rates create gyroscopic couples acting on the satellite in order to control its attitude with respect to the local orbital frame. The new proposed control architecture was software implemented and validated, and the analysis of the obtained results proved the cancellation of the convergence errors and excellent angular rate precision.

## 1. Introduction

The CMG (Control Moment Gyros) type actuators are indispensable drive systems for the controllers of the mini-satellites (satellites under 500 kg), being suitable for fast rotational maneuvers [1,2,3,4,5]. Comparing to the mechanical CMGs, the control moment gyros having rotors with active magnetic bearings (AMB-rotors) and single or double gimbals (SGMSCMGs or DGMSCMGs) possesses the advantages of zero-friction (eliminating the lubrication necessity), of lower noise, of small vibrations, as well as of increased longevity [6,7,8]. There are two kinds of the control moment gyros with two gimbals: (1) magnetically suspended DGCMGs with a magnetically suspended rotor and a stator part that is base-fixed; (2) variable speed double-gimbal control moment gyros (VSDGCMGs) [9]. The DGMSCMGs generate two gyroscopic torques, so a higher cumulative total torque, thus reducing the satellite’s mass and dimensions. An architecture involving a DGMSCMG with AMB allows the decoupling of its translation dynamics from the rotation one, as well as from gimbals’ rotational dynamics; thus, it allows the dynamics’ decoupling, some observers being required for state observation [1,2,10,11,12,13].

An important issue treated by the specialized scientific papers [14,15] is the resonance vibration control; another issue is the one associated with the design of the gimbals’ driving servo systems [7,10,16,17]. To avoid singularities using the null motion, with or without stored energy control, one proposed SGCMG type actuators, as well as actuators based on two or three DGCMGs, in orthogonal or parallel configuration [16,18,19,20,21]. Various satellites’ attitude control systems, based on both gimbals’ angular rates, and gyro-motors angular rates changing control have also been studied [5,15,16,22]. The topic of the satellites’ attitude control using DGMSCMGs, proportional-derivative control laws, proportional-integrator control laws, optimal control laws (with H_2_, H_∞_, H_2_/H_∞_ optimal criteria), gain scheduling control laws (handling the external disturbances such as the coupling gyroscopic effects), integral-terminal-sliding mode control laws, or adaptive control laws, with or without neural networks (NNs), was treated by a lot of scientific papers, such as [1,2,3,9,15,16,23,24,25,26]. The attitude control was improved by means of various control architectures involving the modification of the DGMSCMG gimbals’ angular rates and/or the permanent change of the gyro-motors’ own rotation angular rates [5,27].

There are a lot of drawbacks to the papers mentioned above. The dynamics considered in paper [2] do not consider the rotor-gimbal interactions, the control being not sufficiently precise. The sliding-mode control approach is used to control the satellites’ attitude and to suppress the vibrations in [28], but the drawbacks of this control method involve the mandatory decoupling between the channels associated with the gimbals and the employed coordinate transformation. The parameter perturbation robust control method was employed in [29] to control the dynamics of MSCMGs, the obtained control scheme reaching its critical stability when the rotor angular rate is increased. Paper [19] proposed neural network-based fault diagnosis architectures for the estimation and the fault isolation of the SGCMGs equipping spacecraft by considering multiple actuators (SGCMG type); the attitude control is accurately achieved, but the employed cluster type includes SGCMGs, without decoupling between the rotation and the translation dynamics.

A new dynamics is obtained in work [25] for classical double-gimbal CMG systems, its strong point being the solving of the mismatched problem; also, the unwanted effects of the disturbances are properly cancelled via a backstepping control-based approach. However, this robust control method involves some shortcomings [9]: (a) the dynamics does not consider the rotor dynamics; (b) the nonlinearities of the systems should be completely known or must be estimated with very good accuracy; (c) instead of the gimbal angular rates, the inputs of the controller are the gimbals’ angular displacements. Paper [10] proposed some adaptive control laws to control the translational and rotational dynamics of the AMB-rotor, as well as the control of the gimbals’ angular rates from the component of a DGMSCMG; as the main drawback of this work, we can state the non-consideration of the interactions between the DGMSCMG and the satellite, on which this is placed as actuator and as subsystem of the automatic attitude control architecture.

Different from the above-mentioned control architectures, the present paper aims to design a novel attitude control scheme for mini-satellites by using an actuator with *N* = 2 DGMSCMGs (in parallel/orthogonal configuration) and a DGMSCMG-type sensor for the control of the satellite absolute angular rate. The design of the attitude control architecture is based on the Lyapunov theory. An important element is the actuator, consisting of two DGMSCMGs and a DGMSCMG-type sensor, as part of the actuator-sensor-satellite subsystem. To design the DGMSCMG-based actuator and sensor, each gyroscopic rotor’s translation dynamics was decoupled from its rotation dynamics and from gimbals’ rotation dynamics, followed by the design of the adaptive control of all three subsystems (for the rotors’ translation control, for the rotors’ rotation control, as well as for the gimbals’ rotation control) by using the dynamic inversion concept and neural networks.

The paper is structured as follows: In Section 2, one presents the elements of the attitude calculation approach, based on the quaternion method [30]. In Section 3, one presents the dynamic models of the actuators with *N* = 2 DGMSCMGs, in parallel and orthogonal configurations; the used DGMSCMGs have the architecture described in work [31]. The next section introduces the dynamic model of the DGMSCMG-type sensor for the satellite’s absolute angular rate. Section 5 presents a PD-type control law for the satellite’s attitude control, based on a Lyapunov-type function [32]. In the sixth part of the paper, one presents the results for the performed simulations for both types of actuators (parallel and orthogonal configurations), based on the Simulink-Matlab models.

## 2. Attitude Definition and Calculation

**Figure 1:**
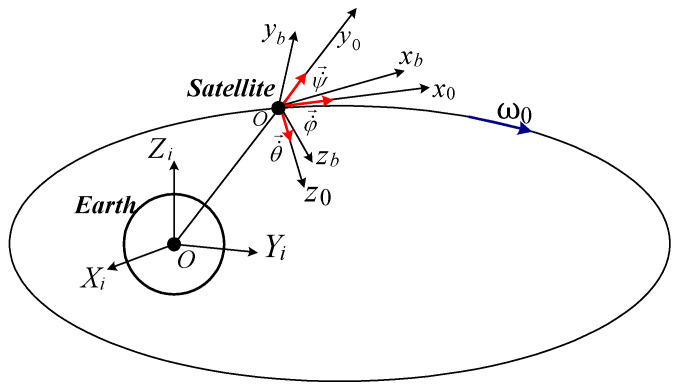
Inertial, local orbital, and satellite-tied frames.

The attitude of the satellite (S), with its tied frame denoted by $Ox_{b}y_{b}z_{b}\;,$ with respect to the local orbital frame $Ox_{0}y_{0}z_{0}$ ($Oy_{0}—$axis oriented in the direction that connects the origin of the initial frame $O_{i}x_{i}y_{i}z_{i}$ to the satellite’s frame origin, $Oz_{0}—$axis oriented in the orbital plan’s anti-normal direction, and $Ox_{0}—$axis completing the orthogonal frame) is given by the Euler angles θ, φ, Ψ (see Figure 1). Rotating successively θ,φ , and Ψ , one obtains the satellite’s frame rotation matrix A , with respect to the local orbital frame, as presented in [17]. In order to avoid the singularities for large Euler angles or a large number of calculations, one uses quaternions for the rotation matrix expression, as in [30]:

(1) $${\left[\begin{array}{cc} q & q_{4} \end{array}\right]}^{T}={\left[\begin{array}{ccc} e_{1}\sin\frac{\phi }{2} & e_{2}\sin\frac{\phi }{2} & e_{3}\sin\frac{\phi }{2}\cos\frac{\phi }{2} \end{array}\right]}^{T},\;q={\left[\begin{array}{ccc} q_{1} & q_{2} & q_{3} \end{array}\right]}^{T}=e\sin\frac{\phi }{2},$$

(2) $$q^{T}q+q_{4}^{2}=1,$$

where ϕ is the satellite’s rotation angle about the Euler axis, $e={\left[\begin{array}{ccc} e_{1} & e_{2} & e_{3} \end{array}\right]}^{T}—$the unit vector on Euler’s axis direction, while $e_{1},e_{2},$ and $e_{3}$ are the vector’s director cosines with respect to the local orbital frame.

Quaternion’s kinematics is defined by the differential equations [30]:

(3a) $$\dot{q}=-\frac{1}{2}\omega_{s}^{\times }q+\frac{1}{2}q_{4}\omega_{s},$$

(3b) $$q_{4}=-\frac{1}{2}\omega_{s}^{T}q,$$

or

(4a) $$\dot{q}=-\frac{1}{2}\left(q^{\times }+q_{4}I_{3}\right)\;\omega_{s},$$

(4b) $$q_{4}=-\frac{1}{2}q\omega_{s},$$

where $\omega_{s}={\left[\begin{array}{ccc} \omega_{sx} & \omega_{sy} & \omega_{sz} \end{array}\right]}^{T}$ is the satellite’s angular rate vector with respect to the local orbital frame (with its components $\omega_{sx},\;\omega_{sy},\;\omega_{sz}$ relative to the axes of the satellite-tied frame $Ox_{b}y_{b}z_{b}$), $I_{3}—\left(3\times 3\right)$ unit matrix,

(5) $$\omega_{s}^{\times }=\left[\begin{array}{ccc} 0 & -\omega_{sz} & \omega_{sy}\\ \omega_{sz} & 0 & -\omega_{sx}\\ -\omega_{sy} & \omega_{sx} & 0 \end{array}\right],\;\omega_{b}^{\times }=\left[\begin{array}{ccc} 0 & -\omega_{bz} & \omega_{by}\\ \omega_{bz} & 0 & -\omega_{bx}\\ -\omega_{by} & \omega_{bx} & 0 \end{array}\right],\;q^{\times }=\left[\begin{array}{ccc} 0 & -q_{3} & q_{2}\\ q_{3} & 0 & -q_{1}\\ -q_{2} & q_{2} & 0 \end{array}\right]\;,$$

with $\omega_{bx},\;\omega_{by},\;\omega_{bz}—$the components of the angular rate vector, relative to the satellite’s axes.

**Figure 2:**
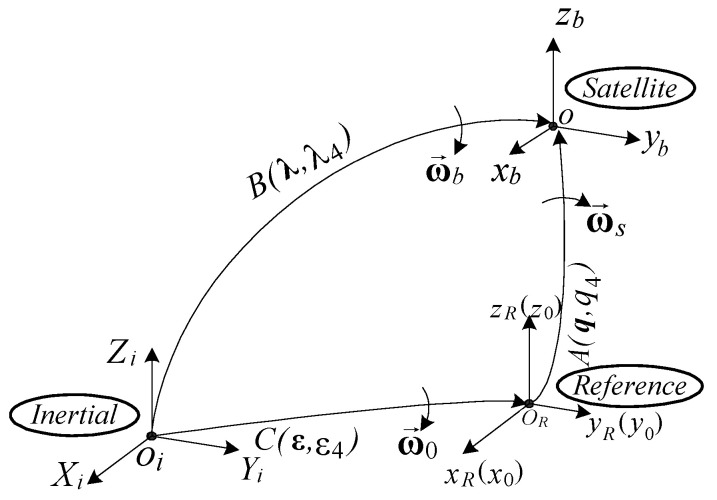
Frames, rotation matrix, and quaternion.

According to Figure 2, one defines the quaternions

(6) $$q={\left[\begin{array}{ccc} q_{1} & q_{2} & q_{3} \end{array}\right]}^{T},\;\lambda ={\left[\begin{array}{ccc} \lambda_{1} & \lambda_{2} & \lambda_{3} \end{array}\right]}^{T},\;\varepsilon ={\left[\begin{array}{ccc} \varepsilon_{1} & \varepsilon_{2} & \varepsilon_{3} \end{array}\right]}^{T},$$

(7) $$q^{T}q+q_{4}^{2}=1\;,\;\;\;\lambda^{T}\lambda +\lambda_{4}^{2}=1,\;\varepsilon^{T}\varepsilon +\varepsilon_{4}^{2}=1,$$

while the rotation matrices are [2]

(8) $$A=A\left(q,q_{4}\right)=\left(q_{4}^{2}-q^{T}q\right)I_{3}+2q^{T}q-2q_{4}q^{\times },$$

(9) $$B=B\left(\lambda ,\lambda_{4}\right)=\left(\lambda_{4}^{2}-\lambda^{T}\lambda \right)I_{3}+2\lambda^{T}\lambda -2\lambda_{4}\lambda^{\times },$$

(10) $$C=C\left(\varepsilon ,\varepsilon_{4}\right)=\left(\varepsilon_{4}^{2}-\varepsilon^{T}\varepsilon \right)I_{3}+2\varepsilon^{T}\varepsilon -2\varepsilon_{4}\varepsilon^{\times },$$

from which it results in the formula B=AC .

The relation between the absolute angular rate $\left(\omega_{b}\right),$ the relative angular rate $\left(\omega_{s}\right),$ and the transport angular rate $\left(\omega_{0}\right)$ is

(11) $$\omega_{b}=\omega_{s}+A\left(q,q_{4}\right)\omega_{0}.$$

If the local orbital frame is considered the reference frame, i.e., $Ox_{R}y_{R}z_{R}\equiv Ox_{0}y_{0}z_{0},$ then $\omega_{0}={\left[\begin{array}{ccc} 0 & 0 & -\omega_{0} \end{array}\right]}^{T}$ and the attitude matrix determined by Equation (8) becomes

(12) $$A=\left[\begin{array}{ccc} q_{1}^{2}-q_{2}^{2}-q_{3}^{2}+q_{4}^{2} & 2\left(q_{1}q_{2}+q_{3}q_{4}\right) & 2\left(q_{1}q_{3}-q_{2}q_{4}\right)\\ 2\left(q_{1}q_{2}-q_{3}q_{4}\right) & -q_{1}^{2}+q_{2}^{2}-q_{3}^{2}+q_{4}^{2} & 2\left(q_{2}q_{3}+q_{1}q_{4}\right)\\ 2\left(q_{1}q_{3}+q_{2}q_{4}\right) & 2\left(q_{2}q_{3}-q_{1}q_{4}\right) & -q_{1}^{2}-q_{2}^{2}+q_{3}^{2}+q_{4}^{2} \end{array}\right]\;.$$

Identifying the elements of the matrix A (θ, φ, Ψ) in work [17] with those associated with matrix *A* in Equation (12), one obtains the Euler angles as follows:

(13) $$\theta =\mathrm{atan}\;\left(-\frac{a_{21}}{a_{22}}\right),\;\theta =\mathrm{atan}\;\left(\frac{a_{23}\cos\Psi }{a_{33}}\right),\;\Psi =\mathrm{atan}\;\left(-\frac{a_{13}}{a_{33}}\right).$$

**Figure 3:**
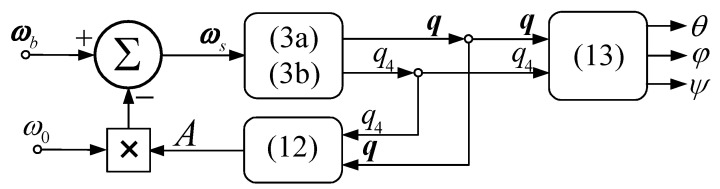
Block diagram of the satellite’s attitude calculation subsystem.

In Figure 3, the block diagram of the satellite’s attitude calculation system is depicted. The components of the satellite’s absolute angular rate $\left(\omega_{bx},\;\omega_{by},\;\omega_{bz}\right)$ are measured by using rate gyros having their sensitivity axes along the axes of the satellite-tied frame, while the components of the satellite’s relative angular rate are computed by means of the block diagram depicted in Figure 3 and by using the formulas.

(14a) $$\omega_{sx}=\omega_{bx}+2\omega_{0}\left(q_{1}q_{3}-q_{2}q_{4}\right)\;,$$

(14b) $$\omega_{sy}=\omega_{by}+2\omega_{0}\left(q_{2}q_{4}+q_{1}q_{3}\right)\;,$$

(14c) $$\omega_{sz}=\omega_{bz}-\omega_{0}\left(q_{1}^{2}+q_{2}^{2}-q_{3}^{2}-q_{4}^{2}\right).$$

## 3. Dynamic Models of the Actuators with *N* = 2 DGMSCMGs

### 3.1. DGMSCMGs with Paralel Architecture

In the satellite’s attitude automatic control system, the subsystem consisting of two DGMSCMGs (in parallel/orthogonal configuration) acts as an actuator. Each one of these reacts at the angular rates ($\omega_{ij}$ and $\omega_{ej},$ where *j* is j=1 for DGMSCMG 1 and j=2 for DGMSCMG 2), computed by the attitude controller and applied to the inner and outer DGMSCMG’s gimbals by means of a servo system.

**Figure 4:**
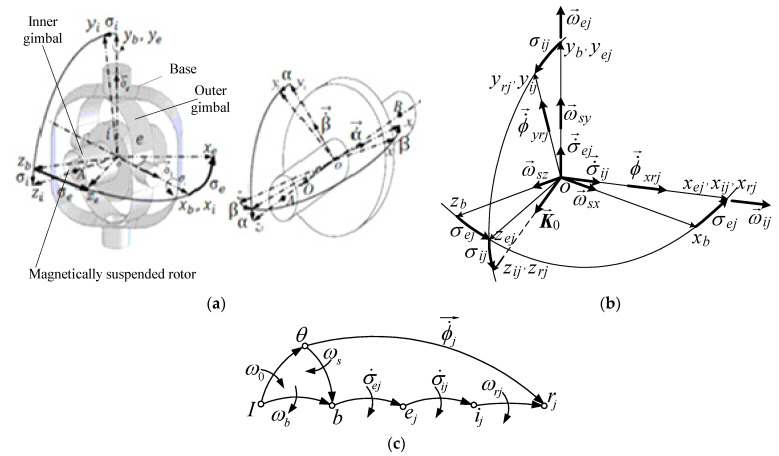
Base-, rotors-, and gimbal-tied frames and angular rates: (**a**) DGSMCMG’s structure; (**b**) gimbals’ angles and angular rates; (**c**) the graph of the interdependences between rotors, gimbals and base.

Figure 4a [31] depicts the simplified DGMSCMG structure (with $j=\bar{1\;,\;2}$); for $\sigma_{i1}=\sigma_{e1}=$ $=\sigma_{i2}=\sigma_{e2}=0\;,$ it is positioned with its inner gimbal axis (*i*) along the $Ox_{b}$ axis, with its outer gimbal axis along the $Oy_{b}$ axis, and with the gyroscopic rotor’s axis (*r*), i.e., the axis of its kinetic couple ${\vec{K}}_{0}\;,$ along the $Oz_{b}$ axis; this way, the outer gimbals are parallel.

Figure 4b depicts the gimbals’ angles and angular rates, the components of the satellite’s relative angular rate vector, as well as the gyroscopic rotors’ precession angular rates (${\vec{\dot{\phi }}}_{xrj}$ and ${\vec{\dot{\phi }}}_{yrj}\;,$ with $j=\bar{1,2}$). Figure 4c depicts the graph of the interdependences between the rotor, the gimbals, and the base, which matches Figure 2. Having in mind that the DGSMCMGs are PD-type automatic control subsystems for the compensation of the linear/angular deviations and rates [10], the angular rate of the gyroscopic rotor can be written relative to the inner gimbal as: ${\vec{\omega }}_{rj}={\vec{\dot{\alpha }}}_{j}+{\vec{\dot{\beta }}}_{j}≅0\;;$ therefore, the frames $Ox_{rj}y_{rj}z_{rj}$ and $Ox_{ij}y_{ij}z_{ij}$ are overlapped and, consequently, the rotation matrix of the rotor-tied frame with respect to the inner gimbal-tied frame becomes $A_{ij}^{rj}≅I_{3}$.

According to Figure 4b,c, the absolute angular rates of the rotor and inner gimbals of the DGSMCMGs relative to the local orbital frame are the resultants of the relative angular rates ${\vec{\dot{\sigma }}}_{i}$ and ${\vec{\dot{\sigma }}}_{c}$ (with respect to the base-tied frame) and the transport angular rate (of the base relative to the local orbital frame) ${\vec{\omega }}_{s}$. Thus, the coordinates of the outer gimbal (*e*) relative to the inner one (*i*) and the base (*b*) are

(15) $${\vec{\dot{\phi }}}_{j}=\vec{{\dot{\sigma }}_{ij}}+\vec{{\dot{\sigma }}_{ej}}+{\vec{\omega }}_{s}=\vec{{\dot{\sigma }}_{ij}}+\vec{{\dot{\sigma }}_{ej}}+{\vec{\omega }}_{sx}+{\vec{\omega }}_{sy}+{\vec{\omega }}_{sz},$$

(16) $${\dot{\sigma }}_{ij}={\left[\begin{array}{ccc} {\dot{\sigma }}_{ij} & 0 & 0 \end{array}\right]}^{T},\;{\dot{\sigma }}_{ej}={\left[\begin{array}{ccc} 0 & {\dot{\sigma }}_{ej} & 0 \end{array}\right]}^{T},\;j=\bar{1,\;2}.$$

Projecting Equation (15) on the axes of the $Ox_{rj}y_{rj}z_{rj}$ frames (overlapped over $Ox_{ij}y_{ij}z_{ij}$) and considering Figure 4b, one yields

(17a) $${\dot{\phi }}_{xrj}≅{\dot{\phi }}_{xij}={\dot{\sigma }}_{ij}+\omega_{sx}\cos\sigma_{ej}+\omega_{sz}\sin\sigma_{ej}\;,$$

(17b) $${\dot{\phi }}_{yrj}≅{\dot{\phi }}_{yij}=\left({\dot{\sigma }}_{ej}+\omega_{sy}\right)\cos\sigma_{ij}-\left(\omega_{sx}\sin\sigma_{ej}-\omega_{sz}\cos\sigma_{ej}\right)\sin\sigma_{ij}\;,$$

where $\omega_{sx},\;\omega_{sy},$ and $\omega_{sz}$ are obtained via Equation (14).

According to Figure 4b, between the angular rates expressed with Equation (17) and the ones provided by the attitude controller, there are the next mathematical relations:

(18) $$\omega_{ij}={\dot{\phi }}_{xrj}≅{\dot{\phi }}_{xij},\;\omega_{ej}={\dot{\phi }}_{yrj}/\cos\sigma_{ij}≅{\dot{\phi }}_{yij}/\cos\sigma_{ij},\;j=\bar{1,2}.$$

By using Equations (17) and (18), one gets the relative angular rates applied to the servo systems driving the gyroscopic gimbals

(19a) $${\dot{\sigma }}_{ije}=\omega_{ij}-\left(\omega_{sx}\cos\sigma_{ej}+\omega_{sz}\sin\sigma_{ej}\right),$$

(19b) $${\dot{\sigma }}_{eje}=\omega_{ej}-\omega_{sy}+\left(\omega_{sx}\sin\sigma_{ej}-\omega_{sz}\cos\sigma_{ej}\right)\;\tan\sigma_{ij}\;,$$

where $\omega_{ij}$ and $\omega_{ej}$ are the components of the algebraic vectors ${\vec{\omega }}_{ij}$ and ${\vec{\omega }}_{ej}\;,$ that, according to Figure 4b, have the following expressions:

(20) $$\omega_{ij}={\left[\begin{array}{ccc} \omega_{ij} & 0 & 0 \end{array}\right]}^{T},\;\omega_{ej}={\left[\begin{array}{ccc} 0 & \omega_{ej} & 0 \end{array}\right]}^{T},\;j=\bar{1,2}.$$

By means of these forms, one can express the matrix

(21) $$\omega_{ij}^{\times }=\left[\begin{array}{ccc} 0 & 0 & 0\\ 0 & 0 & -\omega_{ij}\\ 0 & \omega_{ij} & 0 \end{array}\right],\;\omega_{ej}^{\times }=\left[\begin{array}{ccc} 0 & 0 & \omega_{ej}\\ 0 & 0 & 0\\ -\omega_{ej} & 0 & 0 \end{array}\right]\;.$$

Each of the two DGSMCMGs has the form of the one modeled and designed in paper [10]. Thus, the *j*-th DGSMCMG consists of four subsystems: (1) the automatic control subsystem (with a PID-type control law) for the control of the coordinates $x_{rj}$ and $y_{rj}$ (linear displacements) associated to the gyroscopic rotor; (2) the adaptive control subsystem for the control of the gyroscopic rotor’s angular displacements (consisting of a PD-type dynamic compensator, a linear observer, and a neural network modeling an adaptive control law component); (3) the servo system for the adaptive control of the gyroscopic gimbals’ angular rates ${\dot{\sigma }}_{ij}$ and ${\dot{\sigma }}_{ej}$ (also consisting of a PD-type dynamic compensator, a linear observer, and a neural network for the adaptive control law modeling). The gimbals are driven by electrical motors having their shafts’ axes co-linear with the gimbals’ axes (favorable arranged so that ${\dot{\sigma }}_{ij}$ and ${\dot{\sigma }}_{ej}$ are following the calculated angular rates ${\dot{\sigma }}_{ije}$ and ${\dot{\sigma }}_{eje}\;,$ given by Equation (19)); (4) the subsystem modeling the interaction between the *j*-th DGSMCMG and the satellite.

**Figure 5:**
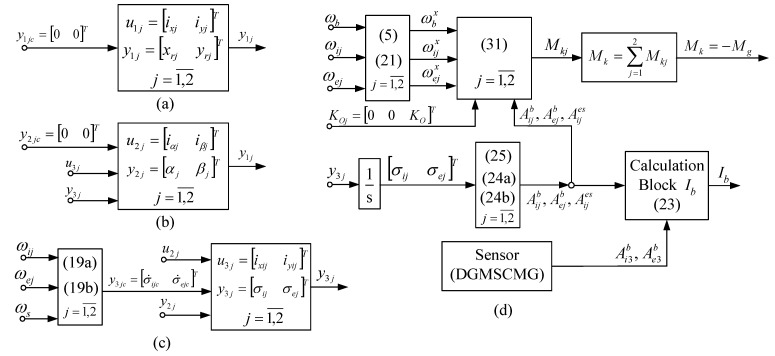
The subsystems of the *j*-th DGMSCMG (**a**) coordinates’ control subsystem; (**b**) gyroscopic rotors’ angular displacements control sub-system; (**c**) gyroscopic gimbals’ angular rates control servo-system and of the actuator with *N* = 2 DGMSCMGs in parallel configuration (**d**).

The first three subsystems, presented in detail in work [10], have simplified forms, depicted in Figure 5a–c, while the last subsystem is presented in Figure 5d. For the first subsystem (Figure 5a), the command vector is $u_{1j}={\left[\begin{array}{cc} i_{xj} & i_{yj} \end{array}\right]}^{T},$ where $i_{xj}$ and $i_{yj}$ are the correction currents, applied to the stator’s coils of the magnetic bearing (two coils in series on the gyroscopic rotor’s axes $Ox_{r}$ and $Oy_{r}$). For the second subsystem (the one in Figure 5b), the command vector is $u_{2j}={\left[\begin{array}{cc} i_{\alpha j} & i_{\beta j} \end{array}\right]}^{T},$ with $i_{\alpha j}$ and $i_{\beta j}—$the correction currents applied to the same coils. For the third subsystem (the one depicted in Figure 5c), the command vector is $u_{3j}={\left[\begin{array}{cc} i_{xi1} & i_{yej} \end{array}\right]}^{T},$ where $i_{xi1}$ and $i_{yej}$ are the currents applied to the command coils of the correction motors.

In Figure 5d, the term $M_{k}=-M_{g}$ represents the input of the satellite’s model, described by the equation:

(22) $$I_{b}{\dot{\omega }}_{b}+\omega_{b}^{\times }I_{b}\omega_{b}=-M_{k}+M_{p},M_{k}=\sum_{j=1}^{2}M_{kj}.$$

In the above formula, $M_{p}$ is the total disturbing moment (applied to the satellite), $I_{b}—$the satellite’s matrix of the inertia moments, considered together with the actuator ($j=\bar{1,2}$) and the angular rate sensor $\left(j=3\right),$ given by the formula in [1]:

(23) $$I_{b}=J_{b}+\sum_{j=1}^{3}\left[{\left(A_{b}^{ej}\right)}^{T}J_{ej}A_{b}^{ej}+{\left(A_{b}^{ij}\right)}^{T}I_{ij}A_{b}^{ij}\right],$$

where $J_{b}$ is the satellite’s matrix of inertia moments (without actuator and sensor), while $J_{ij}$ and $J_{ej}—$are the matrices of the inertia moments for the inner and outer gimbals of the *j*-th DGMSCMG, respectively; $I_{ij}=J_{ij}+J_{rj}$ and $J_{rj}$ are the matrices of inertia moments associated to the gyroscopic rotor, while $A_{b}^{ij}={\left(A_{ij}^{b}\right)}^{T}$ and $A_{b}^{ej}={\left(A_{ej}^{b}\right)}^{T}$ are the rotation matrix of the inner and outer gimbals, related to the satellite base. The components for *j* = 3 are for the DGMSCMG used as the angular rate sensor of the satellite’s base.

For computing the rotation matrices, one can use Figure 4b. The matrices that express the rotation of the outer gimbal relative to the base and the rotation of the inner gimbal with respect to the outer gimbal can be respectively determined as:

(24a) $$\left[\begin{array}{c} x_{ej}\\ y_{ej}\\ z_{ej} \end{array}\right]=A_{b}^{ej}\left[\begin{array}{c} x_{b}\\ y_{b}\\ z_{b} \end{array}\right]=\left[\begin{array}{ccc} \cos\sigma_{ej} & 0 & -\sin\sigma_{ej}\\ 0 & 1 & 0\\ \sin\sigma_{ej} & 0 & \cos\sigma_{ej} \end{array}\right]\left[\begin{array}{c} x_{b}\\ y_{b}\\ z_{b} \end{array}\right],$$

(24b) $$\left[\begin{array}{c} x_{ij}\\ y_{ij}\\ z_{ij} \end{array}\right]=A_{ej}^{ij}\left[\begin{array}{c} x_{ei}\\ y_{ei}\\ z_{ei} \end{array}\right]=\left[\begin{array}{ccc} 1 & 0 & 0\\ 0 & \cos\sigma_{ij} & \sin\sigma_{ij}\\ 0 & -\sin\sigma_{ij} & \cos\sigma_{ij} \end{array}\right]\left[\begin{array}{c} x_{b}\\ y_{b}\\ z_{b} \end{array}\right],$$

(25) $$A_{ij}^{b}≅A_{rj}^{b}={\left(A_{b}^{ij}\right)}^{T}={\left(A_{ej}^{ij}A_{b}^{ej}\right)}^{T},\;A_{ej}^{b}={\left(A_{b}^{ej}\right)}^{T},\;A_{ij}^{ej}={\left(A_{ej}^{ij}\right)}^{T}.$$

According to Figure 4c, the projections of the kinetic moment $K_{0j}={\left[\begin{array}{ccc} 0 & 0 & K_{0} \end{array}\right]}^{T}$ on the axes of the base-tied, on the axes of the inner gimbal-tied frame, and on the axes of the outer gimbal-tied frame have respectively the forms:

(26) $$K_{rb}=A_{rj}^{b}K_{0j},\;K_{rij}=A_{rj}^{ij}K_{0j},\;K_{rej}=A_{rj}^{ej}K_{0j},$$

(27) $$\begin{array}{l} A_{rj}^{b}={\left(A_{b}^{rj}\right)}^{T}={\left(\begin{array}{ccc} A_{ij}^{rj} & A_{ej}^{ij} & A_{b}^{ej} \end{array}\right)}^{T}\approx {\left(\begin{array}{ccc} I_{3} & A_{ej}^{ij} & A_{b}^{ej} \end{array}\right)}^{T}={\left(A_{b}^{ij}\right)}^{T},\;A_{rj}^{ij}≅I_{3},\\ A_{rj}^{ej}={\left(A_{ej}^{rj}\right)}^{T}={\left(\begin{array}{cc} A_{ij}^{rj} & A_{ej}^{ij} \end{array}\right)}^{T}\approx {\left(\begin{array}{cc} I_{3} & A_{ej}^{ij} \end{array}\right)}^{T}={\left(A_{ej}^{ij}\right)}^{T}=A_{ij}^{ej}\;; \end{array}$$

Thus, we write:

(28) $$K_{rb}=A_{ij}^{b}K_{0j},K_{rij}=K_{0j},K_{rej}=A_{rj}^{ej}K_{0j},\;K_{0j}=K_{0},j=\bar{1,2}.$$

The angular rates $\omega_{b},\omega_{ij},\omega_{ej}$ generate the gyroscopic couples:

(29a) $$M_{gb}=K_{rb}^{\times }\omega_{b}=-\omega_{b}K_{rb}^{\times }=-\omega_{b}A_{ij}^{b}K_{0j}=-M_{kb},$$

(29b) $$M_{gij}=K_{rij}^{\times }\omega_{ij}=K_{0j}^{\times }\omega_{ij}=-M_{kij},$$

(29c) $$M_{gej}=K_{rej}^{\times }\omega_{ej}={\left(A_{ij}^{ej}K_{0j}\right)}^{\times }\omega_{ej}=-M_{kej},\;j=\bar{1,\;2}.$$

Projecting these torques on the axes of the $Ox_{b}y_{b}z_{b}$ frame and summing them up, one obtains the following equivalent equations:

(30) $$M_{kj}=-M_{gj}=\omega_{b}^{\times }A_{ij}^{b}K_{0j}-A_{jj}^{b}K_{0j}^{\times }\omega_{ij}-A_{ej}^{b}{\left(A_{ij}^{ej}K_{0j}\right)}^{\times }\omega_{ej},$$

(31) $$M_{kj}=-M_{gj}=\omega_{b}^{\times }A_{ij}^{b}K_{0j}+A_{jj}^{b}\omega_{ij}^{\times }K_{0j}^{\times }+A_{ej}^{b}\omega_{ej}^{\times }\left(A_{ij}^{ej}K_{0j}\right).$$

The total torque created by the two DGMSCMGs of the actuator has one of the following equivalent forms:

(32) $$M_{k}=-M_{g}=\omega_{b}^{\times }\sum_{j=1}^{2}A_{ij}^{b}K_{0j}-\sum_{j=1}^{2}A_{ij}^{b}K_{0j}^{\times }\omega_{ij}-\sum_{j=1}^{2}A_{ej}^{b}{\left(A_{ij}^{ej}K_{0j}\right)}^{\times }\omega_{ej},$$

(33) $$M_{k}=-M_{g}=\omega_{b}^{\times }\sum_{j=1}^{2}A_{ij}^{b}K_{Oj}+\sum_{j=1}^{2}A_{ij}^{b}\omega_{ij}^{v}K_{Oj}+\sum_{j=1}^{2}A_{ej}^{b}\omega_{ej}^{\times }\left(A_{ij}^{ej}K_{Oj}\right).$$

### 3.2. DGMSCMGs with Orthogonal Architecture 

**Figure 6:**
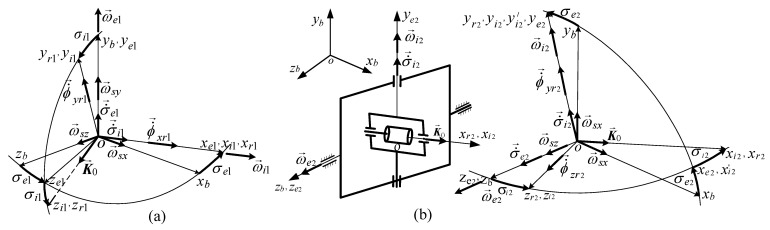
The frames of the DGMSCMG, the rotation angles and the angular rates (**a**) for DGMSCMG 1 and (**b**) for DGMSCMG 2.

The two DGMSCMGs of the actuator have the axes of the outer gimbals perpendicular; DGMSCMG 1 has the axis of the outer gimbal parallel to the $Oy_{b}$ axis (see Figure 4b), while DGMSCMG 2 has the axis of its outer gimbal parallel to $Oz_{b}$ axis. In Figure 6, we present the frames of the rotors, of the inner and outer gimbals, as well as the angular rates of the gyroscopic gimbals for both DGMSCMGs (in Figure 6a for DGMSCMG 1 and in Figure 6b—for DGMSCMG 2).

For DGMSCMG 1, Equations (15)–(25) remains valid, while for DGMSCMG 2, according to Figure 6b, one obtains:

(34a) $${\dot{\phi }}_{yr2}≅{\dot{\phi }}_{yi2}=\omega_{i2}={\dot{\sigma }}_{i2}+\left(-\omega_{sx}\sin\sigma_{e2}+\omega_{sy}\cos\sigma_{e2}\right),$$

(34b) $${\dot{\phi }}_{zr2}≅{\dot{\phi }}_{zi2}=\omega_{e2}\cos\sigma_{i2}=\left({\dot{\sigma }}_{e2}+\omega_{sz}\right)\;\cos\sigma_{i2}+\left(\omega_{sx}\cos\sigma_{e2}+\omega_{sy}\sin\sigma_{e2}\right)\;\sin\sigma_{i2}\;,$$

which lead to new expressions for the calculated angular rates of the gimbals with respect to the base:

(35a) $${\dot{\sigma }}_{i2c}=\omega_{i2}+\left(\omega_{sx}\sin\sigma_{e2}-\omega_{sy}\cos\sigma_{e2}\right),$$

(35b) $${\dot{\sigma }}_{i2c}=\omega_{i2}+\left(\omega_{sx}\sin\sigma_{e2}-\omega_{sy}\cos\sigma_{e2}\right)\;.$$

The expressions of the kinetic moment vector, of the angular rate vector, and of the matrices $\omega_{i2}^{\times },\;\omega_{e2}^{\times }$ are

(36) $$K_{02}={\left[\begin{array}{ccc} K_{0} & 0 & 0 \end{array}\right]}^{T},\;{\dot{\sigma }}_{i2}={\left[\begin{array}{ccc} 0 & {\dot{\sigma }}_{i2} & 0 \end{array}\right]}^{T},{\dot{\sigma }}_{e2}={\left[\begin{array}{ccc} 0 & 0 & {\dot{\sigma }}_{e2} \end{array}\right]}^{T},$$

(37) $$\omega_{i2}={\left[\begin{array}{ccc} 0 & \omega_{i2} & 0 \end{array}\right]}^{T},\;\omega_{e2}={\left[\begin{array}{ccc} 0 & 0 & \omega_{e2} \end{array}\right]}^{T},$$

(38) $$\omega_{i2}^{\times }=\left[\begin{array}{ccc} 0 & 0 & \omega_{i2}\\ 0 & 0 & 0\\ -\omega_{i2} & 0 & 0 \end{array}\right],\;\omega_{e2}^{\times }=\left[\begin{array}{ccc} 0 & -\omega_{e2} & 0\\ \omega_{e2} & 0 & 0\\ 0 & 0 & 0 \end{array}\right].$$

According to Figure 6b,

(39) $$\left[\begin{array}{c} x_{e2}\\ y_{e2}\\ z_{e2} \end{array}\right]=\underset{A_{b}^{e2}}{\underset{︸}{\left[\begin{array}{ccc} \cos\sigma_{e2} & \sin\sigma_{e2} & 0\\ -\sin\sigma_{e2} & \cos\sigma_{e2} & 0\\ 0 & 0 & 1 \end{array}\right]}}\;\left[\begin{array}{c} x_{b}\\ y_{b}\\ z_{b} \end{array}\right],\;\left[\begin{array}{c} x_{i2}\\ y_{i2}\\ z_{i2} \end{array}\right]=\underset{A_{e2}^{i2}}{\underset{︸}{\left[\begin{array}{ccc} \cos\sigma_{i2} & 0 & -\sin\sigma_{i2}\\ 0 & 1 & 0\\ \sin\sigma_{i2} & 0 & \cos\sigma_{i2} \end{array}\right]}}\;\left[\begin{array}{c} x_{e2}\\ y_{e2}\\ z_{e2} \end{array}\right].$$

**Figure 7:**
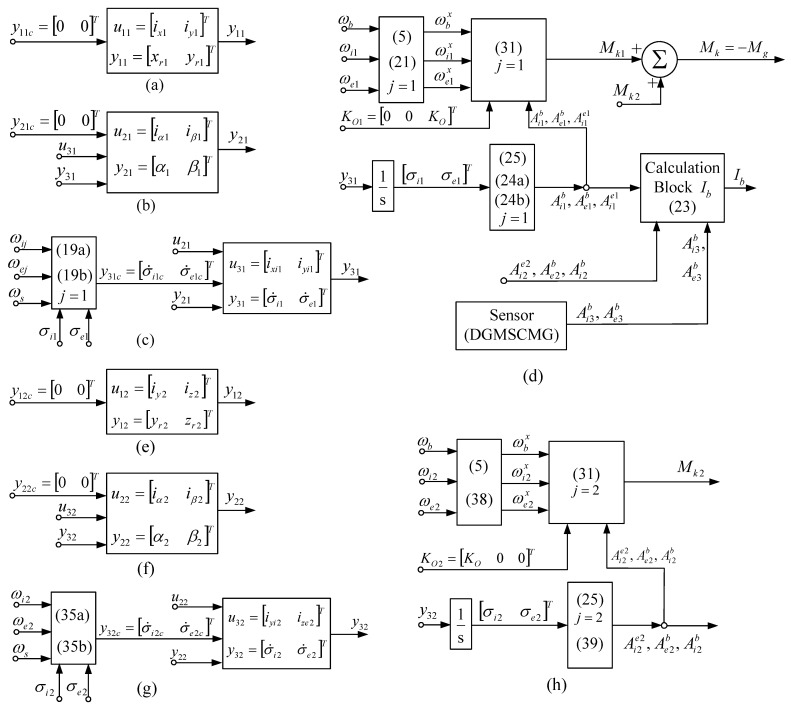
The subsystems of the DGMSCMGs’ models for an actuator with *N* = 2 DGMSCMGs in orthogonal configuration: for DGMSCMG 1 in (**a**–**d**) [(**a**)—coordinates’ control subsystem; (**b**)—gyroscopic rotors’ angular displacements control sub-system; (**c**)—gyroscopic gimbals’ angular rates control servo-system; (**d**)—the subsystem modeling the interaction between DGSMCMG and the satellite] and for DGMSCMG 2 in (**e**–**h**) [(**e**)—coordinates’ control subsystem; (**f**)—gyroscopic rotors’ angular displacements control sub-system; (**g**)—gyroscopic gimbals’ angular rates control servo-system; (**h**)—the subsystem modeling the interaction between DGSMCMG and the satellite].

Equations (25) and (39) are the same for *j* = 2, too. At the same time, one maintains the same calculation formulas (i.e., Equations (30)–(33), for *j* = 1, 2), and also Equation (23). Figure 7 presents the subsystems associated with the DGMSCMGs of the actuator in orthogonal configuration.

## 4. Dynamics of DGMSCMG 3 Sensor for Satellite Absolute Angular Rate Measurement

The disturbed satellite, rotating with $\omega_{s}$ relative angular rate, should be returned to its initial position via an angular rate $-\omega_{s},$ produced by the moment $M_{k}=-M_{g},$ applied by the actuator to the satellite. $M_{k}$ depends only on $\omega_{b}—$the absolute angular rate, in order to return the satellite (aperiodic according to Equation (22)) to its angular rate $\omega_{b}=A\left(q_{s},q_{4s}\right)\omega_{0},$ where $q_{s}$ and $q_{4s}$ denote the stabilized values of the quaternion (33), $M_{k}=M_{k}\left(\omega_{b},\omega_{i},\omega_{e}\right),$ with $\omega_{i}={\left[\begin{array}{cc} \omega_{i1}^{T} & \omega_{i2}^{T} \end{array}\right]}^{T}$ and $\omega_{e}={\left[\begin{array}{cc} \omega_{e1}^{T} & \omega_{e2}^{T} \end{array}\right]}^{T}$. Therefore, the satellite will rotate with a different angular rate ${\hat{\omega }}_{b}\neq \omega_{b};$ thus, an angular rate sensor (DGMSCMG 3) is required for the measurement of ${\hat{\omega }}_{b};$ this sensor is connected to the satellite and to which no angular rates around the outer and inner gimbals’ axes are applied $\left(\omega_{i3}=\omega_{e3}=0\right)$. This sensor is placed in such a way that the axis of its outer gimbal is parallel to the axis of the outer gimbal of DGMSCMG 1.

For the sensor, the Equation (19) becomes (for *i* = 3):

(40a) $${\dot{\sigma }}_{i3c}=-\left(\omega_{sx}\cos\sigma_{e3}+\omega_{sz}\sin\sigma_{e3}\right),$$

(40b) $${\dot{\sigma }}_{e3c}=-\omega_{sy}+\left(\omega_{sx}\sin\sigma_{e3}-\omega_{sz}\cos\sigma_{e3}\right)\;\tan\sigma_{i3}.$$

The sensor is modeled by the equations:

(41) $$M_{ks}=-M_{gs}={\hat{\omega }}_{b}^{\times }A_{i3}^{b}K_{03}=-\left(A_{i3}^{b}K_{03}\right)\;{\hat{\omega }}_{b},\;K_{03}={\left[\begin{array}{ccc} 0 & 0 & K_{0} \end{array}\right]}^{T},$$

(42) $${\hat{\omega }}_{b}=\left[\begin{array}{c} {\hat{\omega }}_{bx}\\ {\hat{\omega }}_{by}\\ {\hat{\omega }}_{bz} \end{array}\right],\;{\hat{\omega }}_{b}^{\times }=\left[\begin{array}{ccc} 0 & -{\hat{\omega }}_{bz} & {\hat{\omega }}_{by}\\ {\hat{\omega }}_{bz} & 0 & {\hat{\omega }}_{bx}\\ -{\hat{\omega }}_{by} & {\hat{\omega }}_{bx} & 0 \end{array}\right]\;.$$

**Figure 8:**
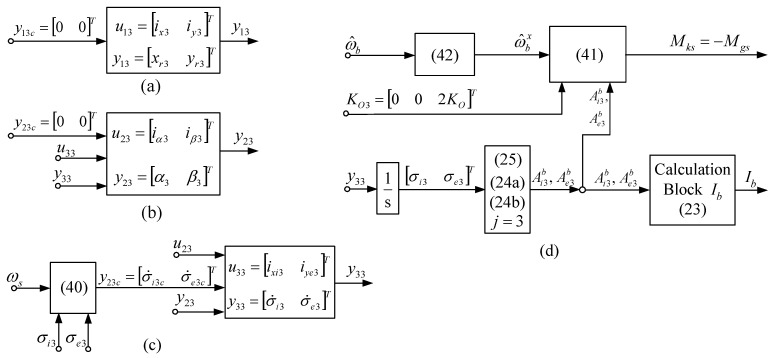
Subsystems of the DGMSCMG 3 used as sensor ) [(**a**)—coordinates’ control subsystem; (**b**)—gyroscopic rotors’ angular displacements control sub-system; (**c**)—gyroscopic gimbals’ angular rates control servo-system; (**d**)—the subsystem modeling the interaction between DGSMCMG and the satellite].

**Figure 9:**
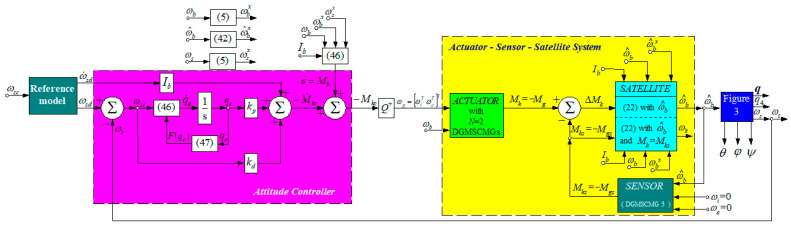
Automatic control system for satellite’s attitude control with PD controller, actuator with *N* = 2 DGMSCMGs, and sensor (DGMSCMG 3).

Consequently, two torques are acting on the satellite: $M_{k}$ and $M_{ks}\;,$ namely $\Delta M_{k}=$ $=M_{k}-M_{ks}$. The subsystems of the DGMSCMG 3 sensor are depicted in Figure 8. As long as the angular rates $\omega_{e}$ and $\omega_{i}$ are null, the system depicted in Figure 9 is stabilized, i.e., $M_{k}=M_{k}\left(\omega_{b}\right)\to M_{ks}\left({\hat{\omega }}_{b}\right)\;,$ which means that $\Delta M_{k}\to 0$ and, thus, ${\hat{\omega }}_{b}\to \omega_{b}\overset{\left(11\right)}{=}A\left(q_{s},q_{4s}\right)\omega_{0}$ and $\omega_{s}\to 0$.

## 5. Satellite’s Attitude Control Using PD-Type Controller

To design the controller, a Lyapunov function is chosen as in [32]:

(43) $$V=\frac{1}{2}\omega_{se}^{T}I_{b}\omega_{se}+2k_{p}\ln\left(1+q_{e}^{T}q_{e}\right),\;k_{p}>0,$$

(44) $$\omega_{se}=\omega_{sd}-\omega_{s},\;q_{e}=M\left(q_{d},q_{4d}\right){\left[\begin{array}{cc} q & q_{4} \end{array}\right]}^{T},$$

where $\omega_{sd}$ is the desired angular rate of the satellite relative to the local orbital frame, $q_{d}—$the desired quaternion of the satellite, q—the quaternion expressing the satellite’s attitude relative to the local orbital frame, $q_{e}—$the error quaternion, while $M\left(q_{d},q_{4d}\right)$ is a matrix having the following form:

(45) $$M\left(q_{d},q_{4d}\right)=\left[\begin{array}{cccc} q_{4d} & q_{3d} & -q_{2d} & -q_{1d}\\ -q_{3d} & q_{4d} & q_{1d} & -q_{2d}\\ q_{2d} & -q_{1d} & q_{4d} & -q_{3d}\\ q_{1d} & q_{2d} & q_{3d} & q_{4d} \end{array}\right].$$

The error quaternion is the solution of the differential equation.

(46) $${\dot{q}}_{e}=F\left(q_{e}\right)\omega_{se},$$

where $F\left(q_{e}\right)$ and $q_{e}$ have the following forms:

(47) $$F\left(q_{e}\right)=\frac{1}{2}\left[\frac{1}{2}\left(1+q_{e}^{T}q_{e}\right)I_{3}+q_{e}^{\times }\right],$$

(48) $$q_{e}=\left[\begin{array}{c} q_{e1}\\ q_{e2}\\ q_{e3} \end{array}\right],\;q_{e}^{\times }=\left[\begin{array}{ccc} 0 & -q_{e3} & q_{e2}\\ q_{e3} & 0 & q_{e1}\\ -q_{e2} & -q_{e1} & 0 \end{array}\right].$$

The time derivative of the Lyapunov function is successively obtained as follows:

(49) $$\begin{array}{ll} \dot{V} & =\omega_{se}^{T}I_{b}{\dot{\omega }}_{se}+\frac{4k_{p}}{1+q_{e}^{T}q_{e}}q_{e}^{T}{\dot{q}}_{e}=\omega_{se}^{T}I_{b}{\dot{\omega }}_{se}+\frac{4k_{p}q_{e}^{T}}{1+q_{e}^{T}q_{e}}F\left(q_{e}\right)\;\omega_{se}\\ & =\omega_{se}^{T}I_{b}{\dot{\omega }}_{se}+\frac{4k_{p}q_{e}^{T}}{1+q_{e}^{T}q_{e}}\frac{1}{2}\left[\frac{1}{2}\left(1+q_{e}^{T}q_{e}\right)\right.I_{3}\left.+q_{e}^{x}\right]\;\omega_{se}\\ & =\omega_{se}^{T}I_{b}{\dot{\omega }}_{se}+k_{p}q_{e}^{T}\omega_{se}+\frac{2k_{p}q_{e}^{T}q_{e}^{\times }}{1+q_{e}^{T}q_{e}}\omega_{se}\\ & =\omega_{se}^{T}I_{b}{\dot{\omega }}_{se}+k_{p}\omega_{se}^{T}q_{e}; \end{array}$$

because $q_{e}^{T}q_{e}^{x}=0\;,$ one yields

(50) $$\dot{V}=\omega_{se}^{T}I_{b}{\dot{\omega }}_{se}+k_{p}\omega_{se}^{T}q_{e}.$$

Imposing $\dot{V}=-k_{d}\omega_{se}^{T}\omega_{se},$ with $k_{d}>0,$ one obtains

(51) $$\dot{V}=\omega_{se}^{T}I_{b}{\dot{\omega }}_{se}+k_{p}\omega_{se}^{T}q_{e}=-k_{d}\omega_{se}^{T}\omega_{se}<0,$$

i.e., the following inequality results:

(52) $$-\omega_{se}^{T}\left(I_{b}{\dot{\omega }}_{se}+k_{d}\omega_{se}+k_{p}q_{e}\right)<0;$$

$\dot{V}<0$ is the stability condition for the closed-loop system depicted in Figure 9.

Replacing ${\dot{\omega }}_{se}$ with its expression ${\dot{\omega }}_{se}={\dot{\omega }}_{sd}-{\dot{\omega }}_{s}$ in the following equation

(53) $$I{\dot{\omega }}_{se}+k_{d}\omega_{se}+k_{p}q_{e}=0\;,$$

one obtains

(54) $$I_{b}{\dot{\omega }}_{sd}+k_{d}\omega_{se}+k_{p}q_{e}=I_{b}{\dot{\omega }}_{s},$$

where the term $I_{b}{\dot{\omega }}_{s}$ comes from the satellite dynamics equation.

The term ${\dot{\omega }}_{b}$ is computed with the derivative of Equation (11), namely: ${\dot{\omega }}_{b}={\dot{\omega }}_{s}+\dot{A}\left(q,q_{4}\right)\;\omega_{0}=$ $={\dot{\omega }}_{s}-\omega_{s}^{x}A\left(q,q_{4}\right)\omega_{0}\overset{\left(12\right)}{=}{\dot{\omega }}_{s}+\omega_{0}\omega_{s}^{x}{\left(col3\right)}_{A};$ introducing it into Equation (22), a new form is obtained:

(55) $$I_{b}{\dot{\omega }}_{s}\overset{\left(22\right)}{=}-M_{k}-\omega_{b}^{\times }I_{b}\omega_{b}-\omega_{0}I_{b}\omega_{s}^{\times }{\left(col3\right)}_{A}+M_{p},$$

where the term ${\left(col3\right)}_{A}$ represents the third column vector of matrix *A*, while $M_{k}$ has the form (32) or (33), i.e.,

(56) $$M_{k}=\omega_{b}^{\times }\sum_{j=1}^{2}A_{ij}K_{0j}-\sum_{j=1}^{2}A_{ij}^{b}K_{0j}^{\times }\omega_{ij}-\sum_{j=1}^{2}A_{ej}^{b}{\left(A_{ij}^{ej}K_{0j}\right)}^{\times }\omega_{ej}=\omega_{b}^{\times }\sum_{j=1}^{2}A_{ij}^{b}K_{0j}+M_{kc},$$

(57) $$M_{kc}=-\sum_{j=1}^{2}A_{ij}^{b}K_{0j}^{\times }\omega_{ij}-\sum_{j=1}^{2}A_{ej}^{b}{\left(A_{ij}^{ej}K_{0j}\right)}^{\times }\omega_{ej}.$$

Invoking Equations (55) and (56), one obtains

(58) $$I_{b}{\dot{\omega }}_{s}=-\omega_{b}^{\times }\left(I_{b}\omega_{b}+\sum_{j=1}^{2}A_{ij}^{b}K_{0j}^{\times }\right)-\omega_{0}I_{b}\omega_{s}^{\times }{\left(col3\right)}_{A}+M_{p}-M_{kc}.$$

One may use the following notations:

(59) $$Q_{ij}=A_{ij}^{b}K_{0j}^{\times },\;Q_{ej}=A_{ej}^{b}{\left(A_{ij}^{ej}K_{0j}\right)}^{\times },\;Q_{j}=\left[\begin{array}{cc} Q_{ij} & Q_{ej} \end{array}\right],\;j=1,2,$$

where $Q_{ij}$ and $Q_{ej}$ are (3 × 3) matrices, while $Q_{j}$ is a (3 × 6) matrix. With these notations, Equation (57) becomes

(60) $$M_{kc}=-\sum_{j=1}^{2}Q_{ij}\omega_{ij}-\sum_{j=1}^{2}Q_{ej}\omega_{ej}=-\sum_{j=1}^{2}\left[\begin{array}{cc} Q_{ij} & Q_{ej} \end{array}\right]\;{\left[\begin{array}{cc} \omega_{ij}^{T} & \omega_{ej}^{T} \end{array}\right]}^{T}=-\sum_{j=1}^{2}Q_{j}\omega_{gj},$$

with

(61) $$Q_{j}=\left[\begin{array}{cc} Q_{ij} & Q_{ej} \end{array}\right],\;\omega_{gj}={\left[\begin{array}{cc} \omega_{ij}^{T} & \omega_{ej}^{T} \end{array}\right]}^{T},\;j=\bar{1,\;2}.$$

The expression (60) might be rewritten as follows:

(62) $$M_{kc}=-Q_{1}\omega_{g1}-Q_{2}\omega_{g2}=-\left[\begin{array}{cc} Q_{1} & Q_{2} \end{array}\right]\;{\left[\begin{array}{cc} \omega_{g1}^{T} & \omega_{g2}^{T} \end{array}\right]}^{Τ}=-Q\omega_{g},$$

with

(63) $$Q=\left[\begin{array}{cc} Q_{1} & Q_{2} \end{array}\right],\;\omega_{g}={\left[\begin{array}{cc} \omega_{g1}^{T} & \omega_{g2}^{T} \end{array}\right]}^{T}={\left[\begin{array}{cc} \omega_{i}^{T} & \omega_{e}^{T} \end{array}\right]}^{T},\;\omega_{i}={\left[\begin{array}{cc} \omega_{i1}^{T} & \omega_{i2}^{T} \end{array}\right]}^{T},\;\omega_{e}={\left[\begin{array}{cc} \omega_{e1}^{T} & \omega_{e2}^{T} \end{array}\right]}^{T}.$$

By using the value of the torque $M_{kc},$ computed by the attitude controller, as well as Equation (62), one obtains

(64) $$\omega_{g}={\left[\begin{array}{cc} \omega_{i}^{T} & \omega_{e}^{T} \end{array}\right]}^{T}=-Q^{+}M_{kc},\;Q^{+}={\left(Q^{T}Q\right)}^{-1}Q^{T},$$

where $Q^{+}$ is the pseudo-inverse of the matrix Q.

Replacing Equation (58) into Equation (54) and using expression (62) for $M_{kc},$ one obtains

(65) $$I_{b}{\dot{\omega }}_{sd}+k_{d}\omega_{se}+k_{p}q_{e}+\omega_{b}^{\times }\left(I_{b}\omega_{b}+\sum_{j=1}^{2}A_{ij}^{b}K_{0j}^{\times }\right)+\omega_{0}I_{b}\omega_{s}^{\times }{\left(col3\right)}_{A}-M_{p}=-M_{kc},$$

or

(66) $$M_{kc}^{/}-{\hat{M}}_{kc}=-M_{kc},$$

where $M_{kc}^{/}$ is the total disturbance moment with expression

(67) $$M_{kc}^{/}=\varepsilon =\omega_{b}^{\times }\left(I_{b}\omega_{b}+\sum_{j=1}^{2}A_{ij}^{b}K_{0j}^{x}\right)+\omega_{0}I_{b}\omega_{s}^{\times }{\left(col3\right)}_{A}-M_{p}$$

and

(68) $$-{\hat{M}}_{kc}=I_{b}{\dot{\omega }}_{sd}+k_{d}\omega_{se}+k_{p}q_{e},$$

This last expression is being modeled by the PD-type attitude controller.

**Remark** **1.**  *The torque $M_{kc}$ is computed for two configurations (orthogonal and parallel), each time with respect to the angular rates (that must be applied to DGMSCMGs’ gimbals) and the rotation matrices associated to the gimbals (relative to the base). By means of the dynamic inversion (i.e., the pseudo-inverse of the matrix Q) and the torque $M_{kc}$ (provided by the attitude controller), the angular rates $\omega_{i}$ and $\omega_{e}$ are computed and the imposed angular rates of the DGMSCMGs’ gimbals are obtained; these angular rates represent the inputs of the servo-systems acting the gimbals and producing the total torque $M_{g}=-M_{k}$ applied to the base in order to obtain its rotation.*

The architecture of the satellite’s attitude controller is depicted in Figure 9. The reference model is a first-order command filter, having the transfer matrix $H_{m}\left(s\right)=\frac{1}{s+1}I_{3}$. It provides both the desired angular rate $\left(\omega_{sd}\right)$ and its time derivative $\left({\dot{\omega }}_{sd}\right);$ the angular acceleration ${\dot{\omega }}_{sd}$ is considered in the control design in order to obtain ${\dot{\omega }}_{s}={\dot{\omega }}_{sd}$.

**Remark** **2.**  *The attitude control of the satellites, *via* the P.D.-type controller, is equivalent to the P.I.-type control of the angular rate $\omega_{s},$ relative to the local orbital frame and, consequently, equivalent to the P.I.-type control of the absolute angular rate $\omega_{b}$. The attitude controller calculates the imposed command torque $M_{kc}$ for the satellite’s orientation and its attitude’s stabilization, based on Lyapunov functions theory. For the satellite’s rotation, the $M_{k}$ torque must be applied; it should tend to $M_{kc}$.*

## 6. Numerical Simulations

**Figure 10:**
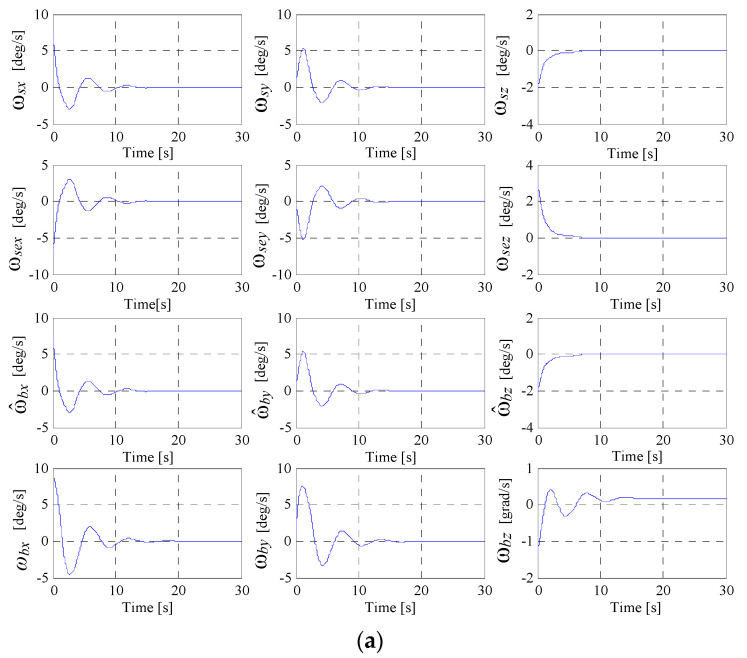

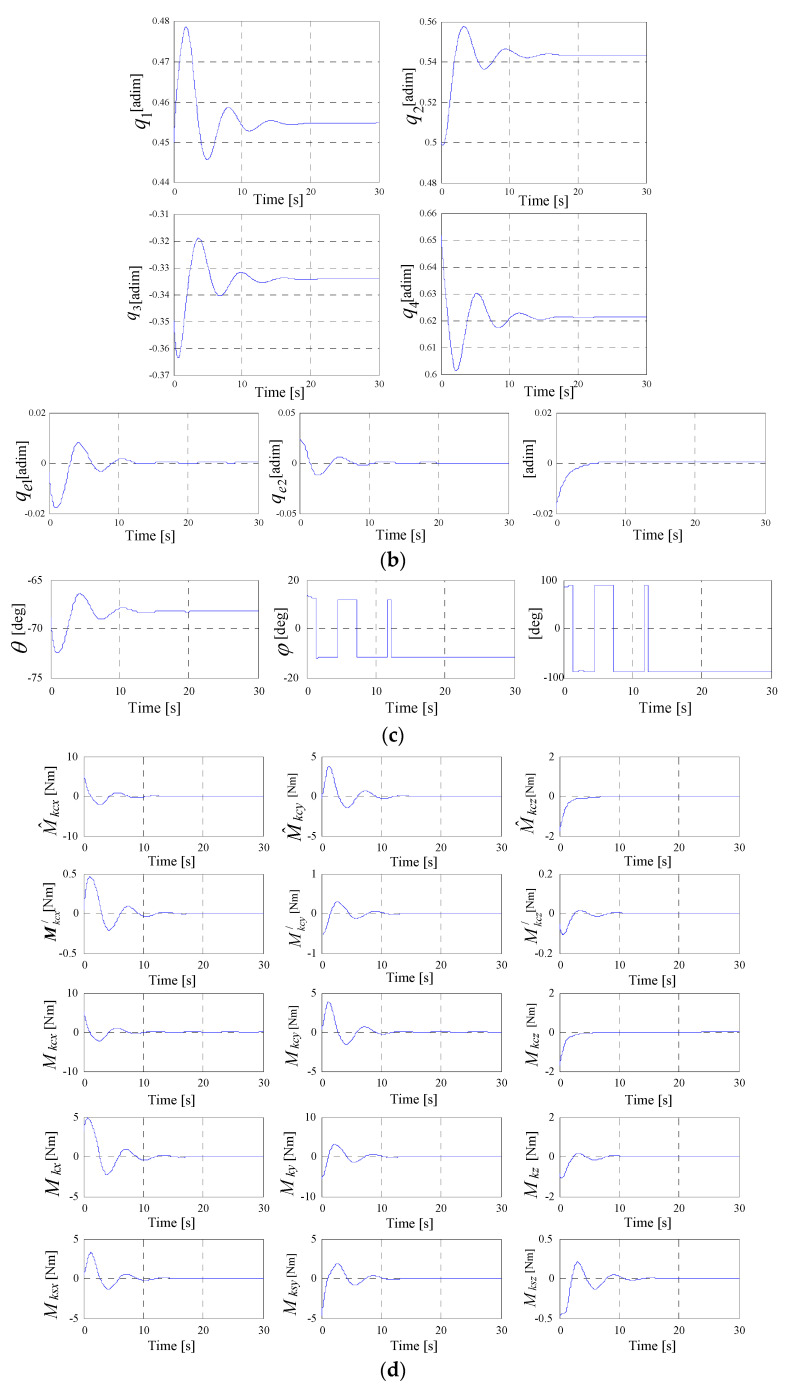

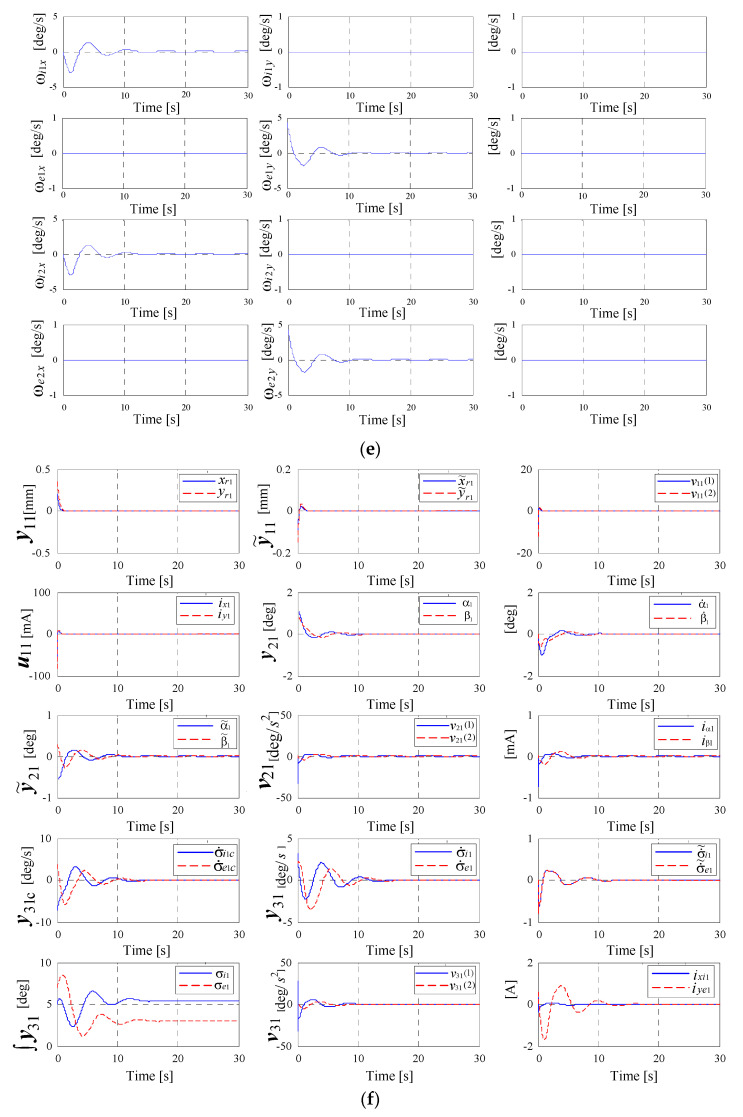

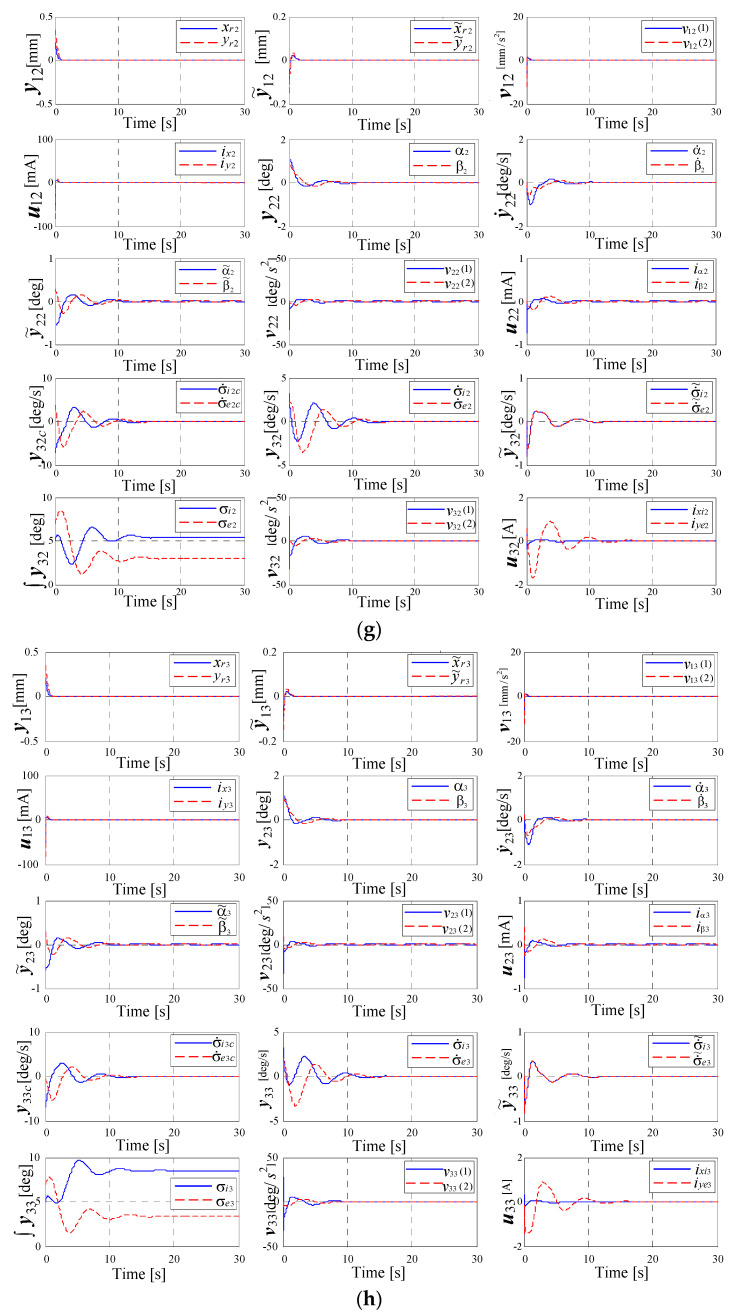
Dynamic characteristics of the system in Figure 9 for an actuator with *N* = 2 DGMSCMGs in parallel configuration: (**a**) the components of the satellite’s relative angular rate vectors, of the error relative angular rate vectors and of the absolute angular rate vectors; (**b**) satellite’s attitude with respect to the local orbital frame; (**c**) the Euler angles; (**d**) components of the torque vectors; (**e**) angular rates applied to the gimbals of the actuator-type DGMSCMG; (**f**) dynamic characteristics of the models of both actuator’s DGMSCMGs; (**g**) dynamic characteristics of the DGMSCMG 3 gyro sensor; (**h**) dynamic characteristics of the DGMSCMG 3 gyro sensor.

**Figure 11:**
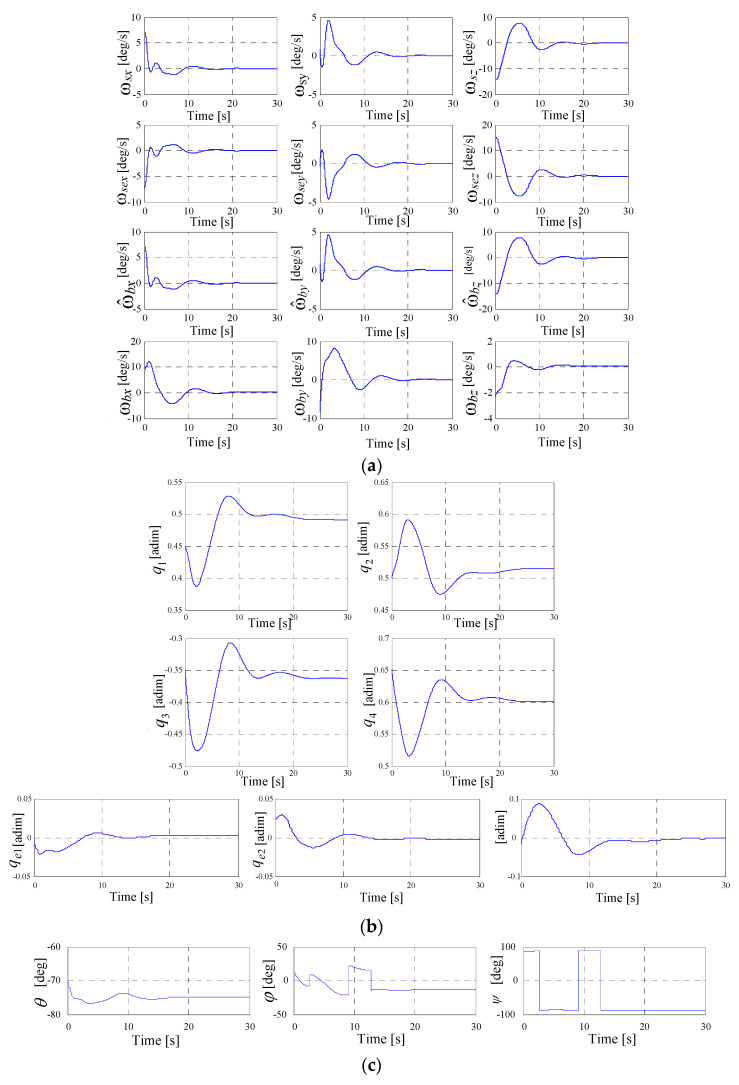

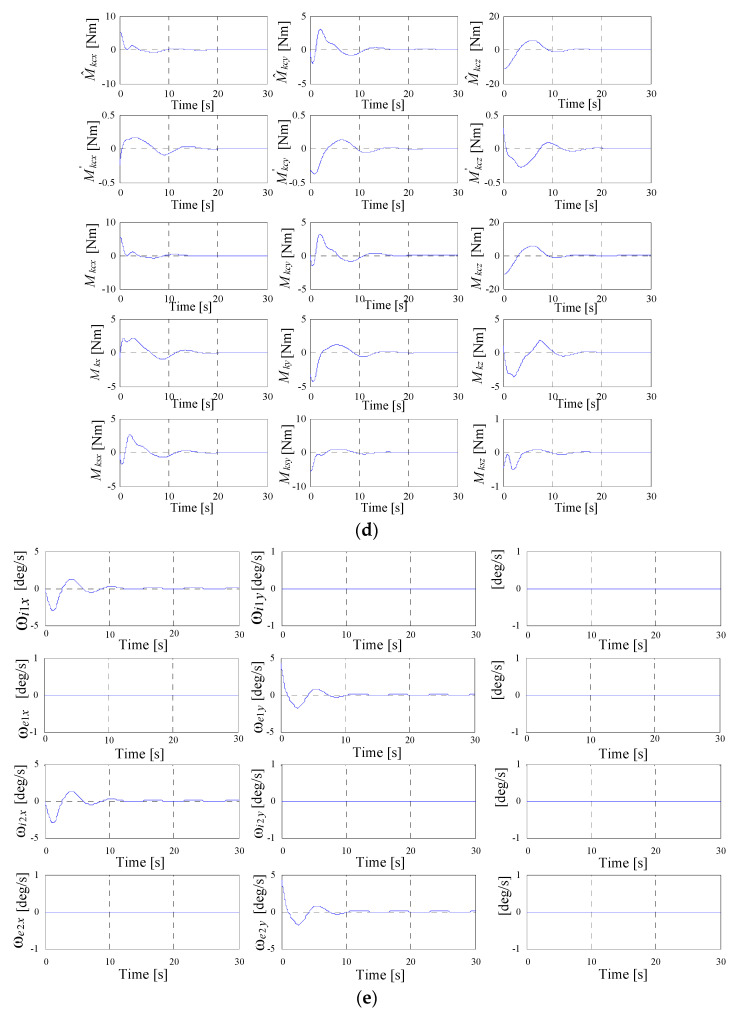

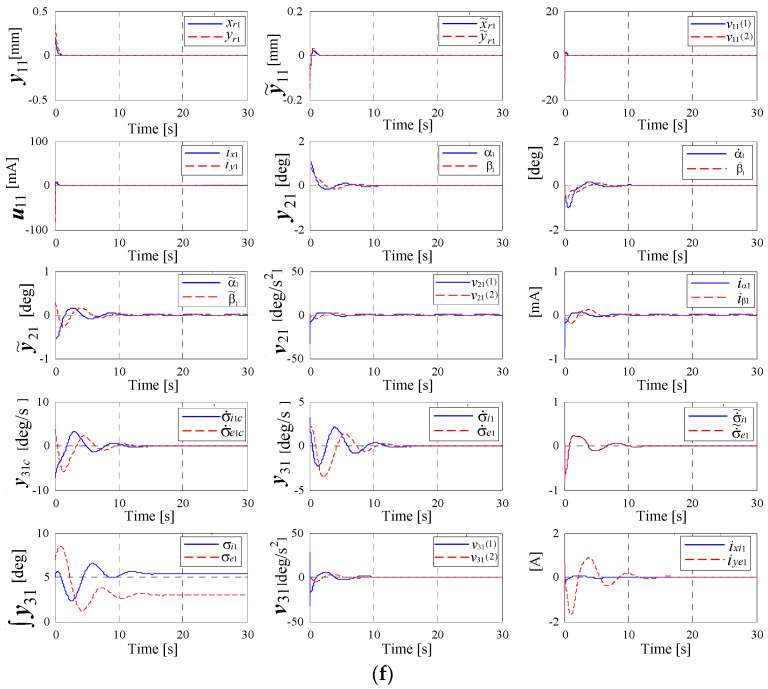

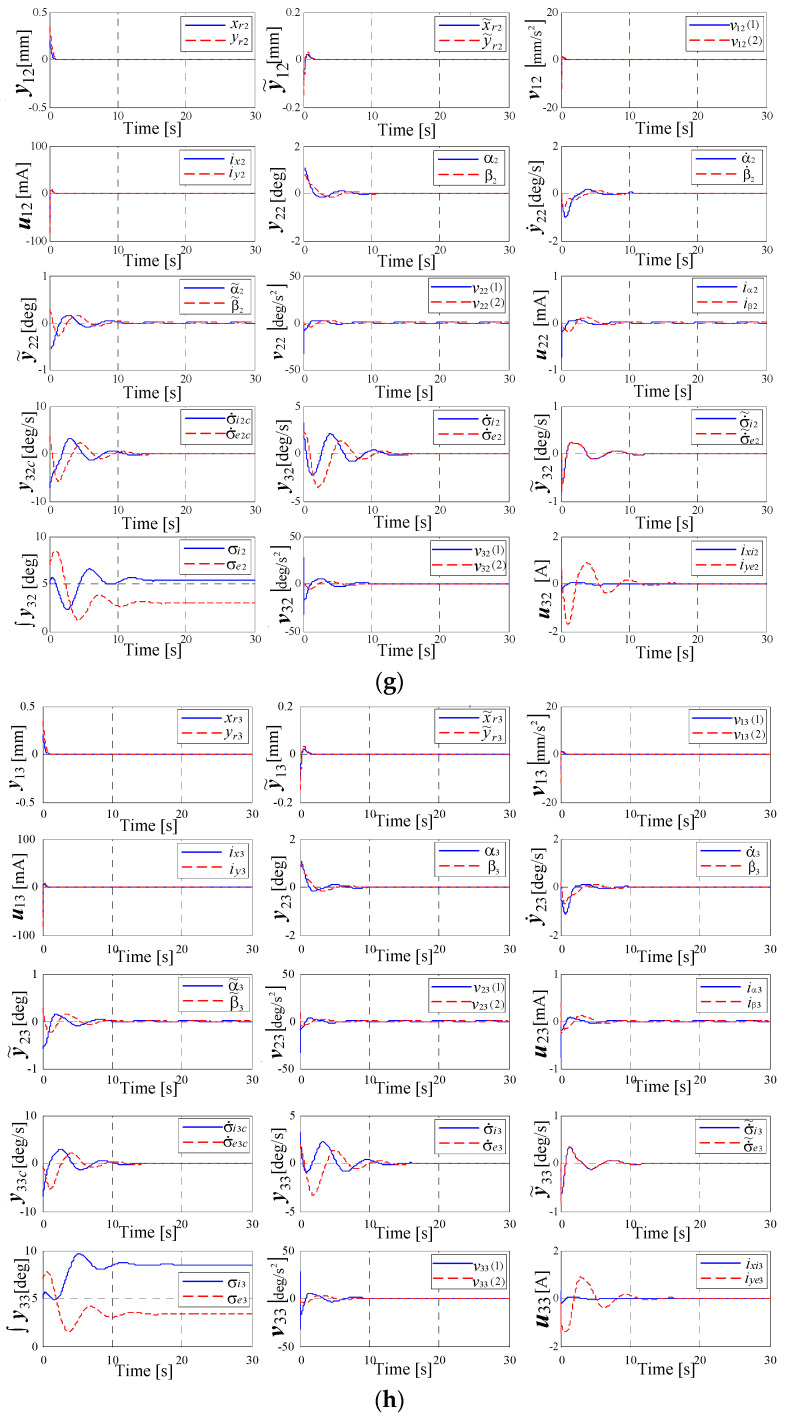
Dynamic characteristics of the system in Figure 9 for an actuator with *N* = 2 DGMSCMGs in orthogonal configuration: (**a**) the components of the satellite’s relative angular rate vectors, of the error relative angular rate vectors and of the absolute angular rate vectors; (**b**) satellite’s attitude with respect to the local orbital frame; (**c**) the Euler angles; (**d**) components of the torque vectors; (**e**) angular rates applied to the gimbals of the actuator-type DGMSCMG; (**f**) dynamic characteristics of the models of both actuator’s DGMSCMGs; (**g**) dynamic characteristics of the DGMSCMG 3 gyro sensor; (**h**) dynamic characteristics of the DGMSCMG 3 gyro sensor.

One has performed a study concerning the dynamics of the system depicted in Figure 9. The numerical values for all three DGMSCMGs are those used in [10] (see Table 1), to which one has added the initial values of the parameters, which might be remarked directly from the graphics in Figure 10 and Figure 11—representing the dynamic characteristics of the system in Figure 9, determined using its Matlab-Simulink model.

The graphics in Figure 10a and in Figure 11a present the components of the satellite’s relative angular rate vector $\omega_{s},$ the components of the error relative angular rate vector $\omega_{se},$ as well as the components of absolute angular rate vectors $\omega_{b}$ and ${\hat{\omega }}_{b}$.

Figure 10b,c and Figure 11b,c present the characteristics expressing the satellite’s attitude with respect to the local orbital frame, namely the quaternion, the error quaternion, and the Euler angles, respectively. Figure 10d and Figure 11d depict the components of the torque vectors ${\hat{M}}_{kc},M_{kc}^{/},M_{kc}$ with respect to the satellite-tied frame, as well as the components of the actuator’s and sensor’s output vectors $\left(M_{k},M_{ks}\right),$ modeled by the systems given in Figure 5d and Figure 7d, respectively.

Figure 10e and Figure 11e present the characteristics, $\omega_{ij},\;\omega_{ej},\;j=\bar{1\;,2},$ i.e., the angular rates applied to the gimbals of the actuator-type DGMSCMGs. In Figure 10f and in Figure 11f, there are graphically represented the dynamic characteristics of the models of both actuator’s DGMSCMGs, shown in a simplified manner in Figure 5a,b,c and Figure 7a,g, respectively, having their complete structure provided in work [10]. Thus, for each DGMSCMG of the actuators, one has represented:

- the components of the vectors of the linear displacements associated to the rotors of the gyro-motors, with respect to the precession axes, i.e., $y_{1j},\;j=\bar{1,2};$
- the components of the error vectors ${\tilde{y}}_{1j},$ with ${\tilde{y}}_{1j}=y_{1c}-y_{1j},\;j=\bar{1,2};$
- the components of the pseudo-command vectors for the control of the gyroscopic rotor’s linear displacements, namely $V_{1j},\;j=\bar{1,2};$
- the components of the command vectors $u_{1j},\;j=\bar{1,2},$ i.e., the currents applied to the correction stator coils of the gyroscopic rotors’ magnetic bearings;
- the components of the vectors $y_{2j},\;{\dot{y}}_{2j},\;{\tilde{y}}_{2j},\;j=\bar{1,2},$ associated with the gyroscopic rotors, namely the vectors consisting of the precession angles, the precession angular rates, and the precession angles’ errors, respectively;
- the components of the control vectors $u_{2j},\;j=\bar{1,2},$ and the components of the pseudo-command vectors $V_{2j},\;j=\bar{1,2},$ all of them associated with the subsystems for the adaptive control of the precession angles; the vectors $u_{2j},\;j=\bar{1,2},$ contain the command currents applied to the same coils of the bearings;
- the components of the vectors $y_{31jc}$ (calculated angular rates), $y_{31j}$ (rotation angular rates), ${\tilde{y}}_{31j}$ (error angular rates), and $y_{31ij}$ (rotation angles of the gimbals);
- the components of the vectors $V_{3j}$ and $u_{3j},\;j=\bar{1,2},$ associated to the command servo systems for the gimbals for the two DGMSCMGs; $u_{3j}$ contain the currents applied to the command coils of the correction motors on the inner and outer gimbals.

In the Figure 10g and Figure 11g, there are represented the dynamic characteristics of the DGMSCMG 3 gyro sensor, characteristics that are similar to those in Figure 10f and Figure 11f, obtained for the DGMSCMGs belonging to the actuator.

Comparing the dynamic characteristics of the attitude control system with *N* = 1 DGMSCMG to those of the attitude control system with *N* = 2 DGMSCMGs (in parallel/orthogonal configuration), one remark is that the architectures with actuators having *N* = 1 DGMSCMG have slightly higher overshoots, are more oscillating, and are a little bit slower.

**Remark** **3.**  *Gyroscopic actuators with N DGMSCMGs (N = 1, 2, or 3), in orthogonal or parallel configuration, compared to similar DGVSCMG-based actuators [8,15,33,34], have the advantage of zero friction in their gyroscopic bearings, which makes unnecessary both the lubrication and the control of the rotors’ speed(s) and stored kinetic energy; thus, they have superior performances. However, DGMSCMGs must be equipped with systems for the automatic control of the gyroscopic rotors’ linear and angular displacements.*

**Remark** **4.**  *In some papers approaching the same topic, the control of DGMSCMGs’ dynamics is based on the decoupling of the rotors’ translation dynamics from their rotation dynamics. For the control of rotors’ and gimbals’ nonlinear rotation dynamics, one has used differential geometry’s principles [11,12]; in the present paper, the control is based on the coupling of the gyroscopic rotors’ rotation dynamics to the gimbals’ rotation dynamics. Comparing the dynamic and static characteristics of the described actuators’ structures (with N = 2 DGMSCMGs in orthogonal/parallel configurations) using the differential geometry’s principles [11,12] to those in the present paper (using the dynamic inversion concept and the adaptive control based on neural networks—a novel element in the field of gyro-systems), the superiority of the latter is noteworthy. This second control technique leads to superior and less oscillating dynamic characteristics, with smaller overshoot, convergence time, and static errors; that means superior performances, especially from the precision of the satellite’s attitude orientation/stabilization point of view.*

An important novelty element in this paper (the use of a supplementary DGMSCMG-type sensor on the actuator’s feedback) contributes to the improvement of the above-mentioned performances. The gyroscopic torque generated by this sensor and applied to the satellite compensates the positioning error of the satellite due to the error of the satellite’s angular rate canceling during its stabilization.

Another advantage of the above-described control architecture is related to the adaptive control law, which uses a small number of sensors, namely sensors for gyroscopic rotors’ total linear displacements and for gimbals’ angular rates, respectively.

## 7. Conclusions

This work first introduced some calculation elements concerning the attitude control of the mini-satellites by using the quaternion theory and Euler angles. Then, there are presented some models for two architectures of the actuator, with *N* = 2 DGMSCMGs, in parallel and orthogonal configuration, additionally considering the third DGMSCMG as a sensor for the measurement of the satellite’s absolute angular rate $\left(\omega_{b}\right)$. One has calculated the total gyroscopic torques created by the two DGMSCMGs of the actuator relative to the angular rate $\omega_{b}\;,$ the angular rates $\omega_{ij},\;\omega_{ej},\;j=\bar{1,2}$ (computed by the attitude controller and applied to the servo-systems belonging to the DGMSCMGs for the actuation of the gimbals), as well as the kinetic torques induced to the satellite by the kinetic moments of the gyroscopic rotors and gimbals. A novel automatic attitude control system has been obtained, its main subsystem being a PD-type controller. For this adaptive control scheme, by means of complex simulations in the Matlab-Simulink environment, one has obtained the dynamic characteristics for both architectures (the one with two DGMSCMGs in parallel configuration and the one with two DGMSCMGs in orthogonal configuration).

## Figures and Tables

**Table 1:** Parameters of the DGMSCMG.

Parameter	Value	Parameter	Value	Parameter	Value	Parameter	Value
*m* [kg]	2.8	*J_iz_* [kg·m^2^]	$2\times 10^{-2}$	*J_ry_* [kg·m^2^]	$45\times 10^{-3}$	*k_xr_* [N/A]	0.21
*l_m_* [m]	$4.1\times 10^{-2}$	*J_ey_* [kg·m^2^]	$15\times 10^{-2}$	*J_rz_* [kg·m^2^]	$65\times 10^{-3}$	*k_yr_* [N/A]	0.21
*l_s_* [m]	$6.5\times 10^{-2}$	*k_hx_* [N/m]	0.8	*J_ix_* [kg·m^2^]	$2\times 10^{-2}$	*k_xi_* [Nm/A]	$3\times 10^{-3}$
*J_rx_* [kg·m^2^]	$45\times 10^{-3}$	*k_hy_* [N/m]	0.8	*J_iy_* [kg·m^2^]	$2\times 10^{-2}$	*k_ye_* [Nm/A]	$2\times 10^{-3}$

## Data Availability

The data presented in this study are available on request from the corresponding author.
